# LigaSure versus Conventional Parotidectomy: A Systematic Review and Meta-Analysis

**DOI:** 10.3390/healthcare10040706

**Published:** 2022-04-11

**Authors:** Sonia Wei-Ting Chen, Li-Jen Hsin, Wan-Ni Lin, Yao-Te Tsai, Ming-Shao Tsai, Yi-Chan Lee

**Affiliations:** 1 Department of Otolaryngology—Head and Neck Surgery, Chang Gung Memorial Hospital, Keelung 20401, Taiwan; b9802057@cgmh.org.tw; 2 College of Medicine, Chang Gung University, Taoyuan 33302, Taiwan; lijen.hsin@gmail.com (L.-J.H.); y1829@cgmh.org.tw (W.-N.L.); s881057@cgmh.org.tw (Y.-T.T.); b87401061@cgmh.org.tw (M.-S.T.); 3 Department of Otolaryngology—Head and Neck Surgery, Chang Gung Memorial Hospital, Taoyuan 33305, Taiwan; 4 Department of Otolaryngology—Head and Neck Surgery, Chang Gung Memorial Hospital, Chiayi 61363, Taiwan

**Keywords:** parotidectomy, parotid surgery, bipolar vessel sealing system, LigaSure

## Abstract

Surgery with parotidectomy is the preferable treatment for most parotid tumors. Our meta-analysis compared the differences between the use of the LigaSure (LS) device and the conventional suture ligation technique (CT) in parotidectomies. A literature search in databases including EMBASE, MEDLINE, and the Cochrane Library was carried out. Studies including parotidectomy using LS and CT were included with the intraoperative and postoperative parameters collected. Continuous operative time data were measured by mean differences (MDs). Discrete data on postoperative complications, including facial palsy, postoperative bleeding, and salivary complications, were evaluated with risk differences (RDs). All values were reported with 95% confidence intervals (CIs). Five studies were included in our meta-analysis. The pooled analysis demonstrated a significant reduction in operative time in the LS group (MD: −21.92; 95% CI, −30.18 to −13.66). In addition, the analysis indicated that the incidence of postoperative complications, including permanent facial palsy (RD, −0.01; 95% CI, −0.06 to 0.05), temporary facial palsy (RD, 0.00; 95% CI, −0.03 to 0.04), salivary complications (RD, −0.01; 95% CI, −0.08 to 0.06), and postoperative bleeding (RD, −0.02; 95% CI, −0.07 to 0.04), were all similar between the LS group and the CT group. According to the results, the LS device appears to be a safe and useful tool and could shorten the operative time in patients needing parotidectomy.

## 1. Introduction

Parotid tumors are the most common salivary gland tumors, accounting for 80% of salivary neoplasms [1,2]. Surgical treatment with parotidectomy is the preferable treatment for most parotid tumors.

Meticulous dissection and facial nerve protection are both crucial procedures in parotidectomy. Suture ligation, cold instruments, and electrocoagulation are conventionally used in surgical practice [3]. In recent years, various energy-based devices have been proposed to facilitate the ligation step in modern surgery [4]. LigaSure (LS), a bipolar vessel sealing system, is one of these energy-based instruments that permanently seals vessels and connective tissue effectively [5]. It also confines the energy transmission within the device, resulting in a thermal spread of less than 2 mm [6], which minimizes the thermal injury to surrounding tissues [7]. LS was reported to reduce operative time and perioperative blood loss in several types of surgery [8,9,10]. The use of LS in parotidectomies, however, has not been reviewed systemically.

The purpose of this study was to collect the available English language literature and analyze the parameters both during and after the operation between parotidectomy using LS and the conventional technique (CT).

## 2. Methods

### 2.1. Literature Search

We carried out systematic literature research in the EMBASE, MEDLINE, and Cochrane Library databases with no limits on article types or date. We retrieved studies using the following headings and keywords: “parotid gland surgery”, “parotidectomy”, “LigaSure”, and “bipolar vessel sealing device”. We searched for studies published in English reporting the use of LS in adult human patients receiving parotidectomy.

### 2.2. Study Selection and Data Extraction

Two authors (S.W.-T.C. and Y.-C.L.) independently screened all the obtained articles by title and abstract. The authors reviewed all the potentially interesting articles by full text. Five articles that met our inclusion and exclusion criteria were eventually included in our study [11,12,13,14,15]. From each study, we extracted data including author, year of publication, sample size, patient age, sex, surgical technique, operation time, and postoperative complications. The CT described in the present study means that the dissection and hemostasis step during surgery was achieved with cold instruments, suture ties, or electrocautery, such as monopolar and bipolar electrocautery. Studies using devices such as a harmonic scalpel or Thunderbeat^®^ (Olympus, Tokyo, Japan) were not included in the present analysis. The LS group, on the other hand, means that LS was used in the surgical steps in parotidectomy.

### 2.3. Risk of Bias Assessment

The risk of bias of the included nonrandomized studies was appraised by using the Newcastle–Ottawa Scale Quality Assessment. Cochrane risk of bias assessment version 1.0 was applied to assess the included randomized studies.

### 2.4. Data Analysis

Mean differences (MDs) were computed to assess the time of operation. Risk differences (RDs) were applied to estimate the occurrence rate of postoperative complications, including facial palsy, postoperative bleeding, and salivary complications (fistula or seroma). Values were reported with 95% confidence intervals (CIs). The overall effect was calculated using a random-effects model. We calculated the *I*^2^ test to assess statistical heterogeneity across the pooled studies. Egger’s tests and visual inspection of funnel plots were used to appraise the publication bias of the included studies [16].

## 3. Results

### 3.1. Study Selection

**Figure 1:**
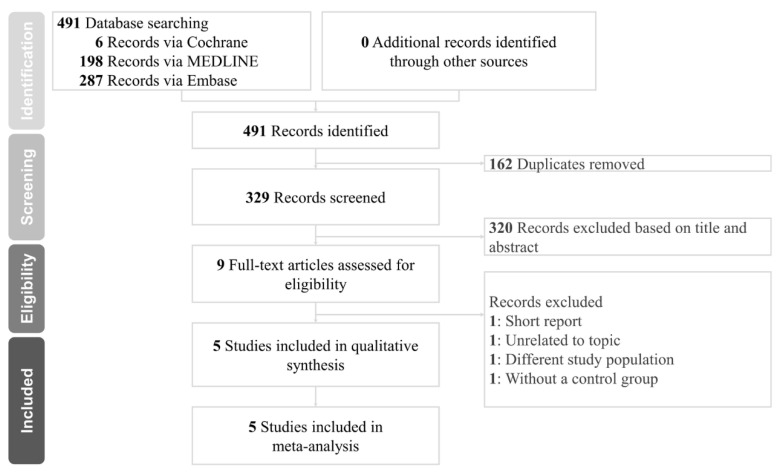
Flow diagram of the literature search.

The literature research step initially located 491 articles in total. After duplicates and 320 articles were eliminated following a check of their titles and abstracts, the remaining nine articles were obtained for a detailed full-text inspection. Among these, articles that were review articles/short reports, studies with no control group, studies involving nonparotidectomy patients, and studies with unclear inclusion were excluded. Eventually, five studies were included in the pooled analysis [11,12,13,14,15]. A flow chart illustrating the process of the literature search, study identification, and inclusion/exclusion criteria is presented in Figure 1. The keywords are summarized in Table S1 of the Supplementary Material.

### 3.2. Demographics

Table 1 classifies demographic data of patients from the five included studies, including four prospective studies and one retrospective study. Among the five studies, three studies used LigaSure Precise (LSP) [12,14,15], and two studies used LigaSure Small Jaw (LSJ) [11,13]. The two types of vessel-sealing instruments were both included in the pooled analysis. The quality and bias assessment for the included studies is disclosed in Tables S2 and S3 in the Supplementary Material. The PRISMA checklist is contained in Table S4 of the Supplementary Material.

### 3.3. Outcomes

#### 3.3.1. Operative Duration

**Figure 2:**
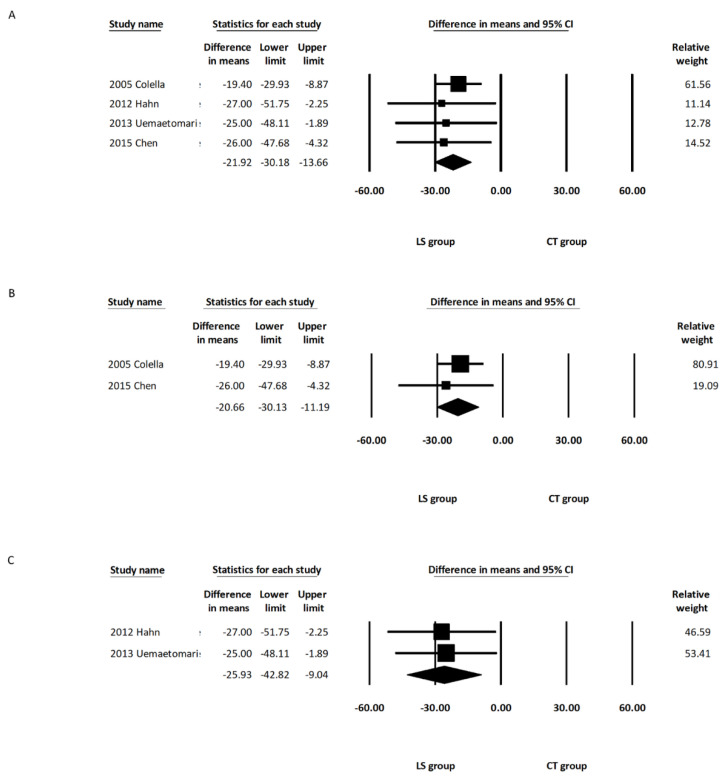
Forest plot of the operative duration during parotidectomy. (**A**) Overall study group. (**B**) Studies including only the LSP device. (**C**) Studies including only the LSJ device. LS, LigaSure; LSP, LigaSure Precise; LSJ, LigaSure Small Jaw; CT, conventional technique; CI confidence interval.

Four of the included studies [11,13,14,15] reported the operative duration of both groups. The pooled analysis demonstrated a significant intergroup difference regarding the operative duration (MD: −21.92; 95% CI, −30.18 to −13.66, *I*^2^ = 0.00%) (Figure 2A). A reduction of 21.92 min was identified in the overall LS group. Subgroup analysis including the two studies using LSP [14,15] revealed a significant reduction in operative duration in the LSP group compared with the CT group (MD: −20.66; 95% CI, −30.13 to −11.19, *I*^2^ = 0.00%) (Figure 2B). Subgroup analysis including the other two studies using LSJ [11,13] also showed a significant reduction in operative duration in the LSJ group compared with the CT group (MD: −25.93; 95% CI, −42.82 to −9.04, *I*^2^ = 0.00%) (Figure 2C). One of the five studies included deep lobe parotid tumors in their report, and the reduced operative duration was still observed in the LS group after removing this study from the analysis (MD: −27.41; 95% CI, −30.32 to −12.62, *I*^2^ = 0.00%) (Figure S1A of the Supplementary Material). Random effects meta-analysis was also calculated with the only one randomized controlled trial excluded [14]. There were no marked differences in the direction or significance of our findings when this study was excluded (Figure S2A of the Supplementary Material).

#### 3.3.2. Postoperative Facial Palsy

Four studies reported the incidence of permanent facial palsy [11,13,14,15], and five studies reported the incidence of temporary facial palsy [11,12,13,14,15]. Meta-analyses were carried out in both types of facial nerve palsy.

**Figure 3:**
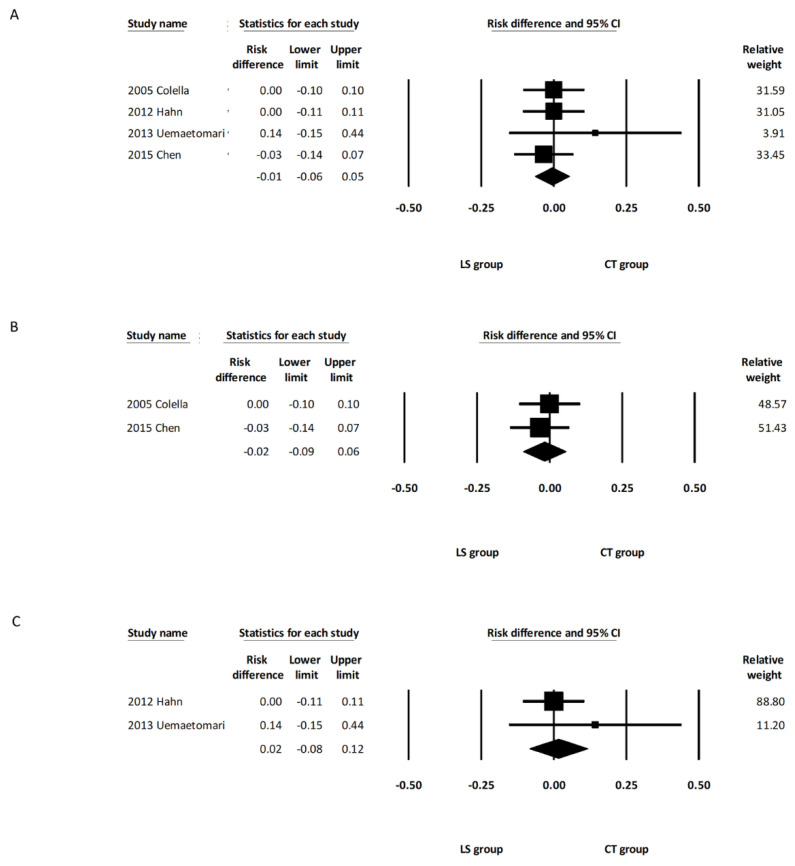
Forest plot of the incidence of permanent facial palsy after parotidectomy. (**A**) Overall study group. (**B**) Studies including only the LSP device. (**C**) Studies including only the LSJ device. LS, LigaSure; LSP, LigaSure Precise; LSJ, LigaSure Small Jaw; CT, conventional technique; CI, confidence interval.

The pooled analysis showed that the incidence of permanent facial palsy was comparable between the overall LS and CT groups (RD, −0.01; 95% CI, −0.06 to 0.05, *I*^2^ = 0.000%) (Figure 3A). Subgroup analysis including the two studies using LSP [14,15] revealed that the incidence of permanent facial palsy was comparable between the LSP group and CT group (RD: −0.02; 95% CI, −0.09 to 0.06, *I*^2^ = 0.00%) (Figure 3B). Subgroup analysis including the two studies using LSJ [11,13] also revealed that the incidence of permanent facial palsy was comparable between the LSJ group and CT group (RD: 0.02; 95% CI, −0.08 to 0.12, *I*^2^ = 0.00%) (Figure 3C).

**Figure 4:**
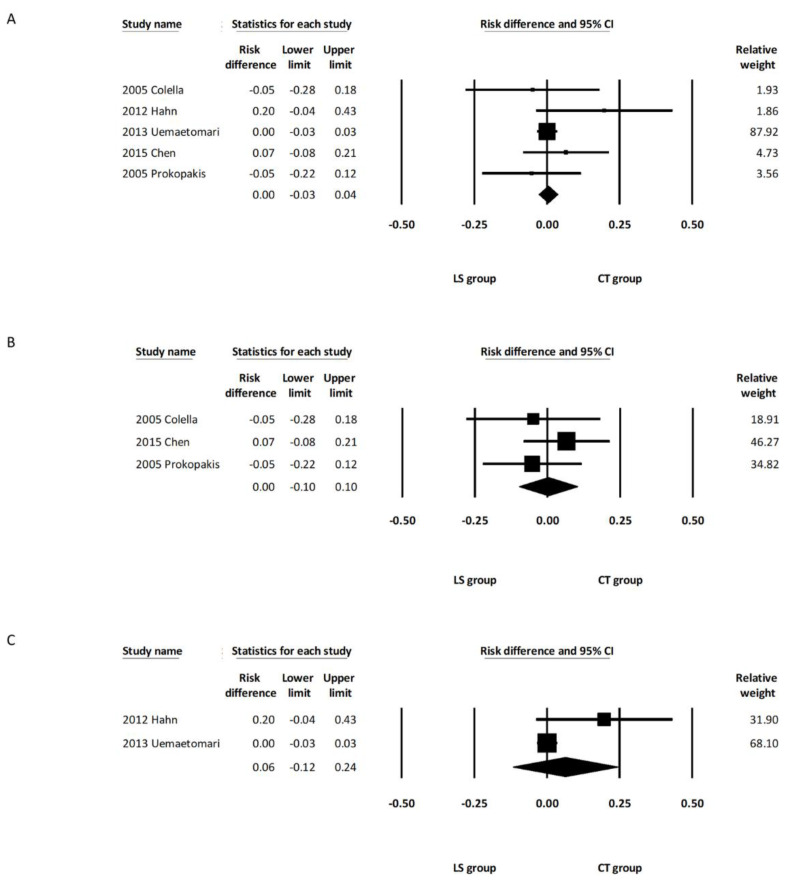
Forest plot of the incidence of temporary facial palsy after parotidectomy. (**A**) Overall study group. (**B**) Studies including only the LSP device. (**C**) Studies including only the LSJ device. LS, LigaSure; LSP, LigaSure Precise; LSJ, LigaSure Small Jaw; CT, conventional technique; CI confidence interval.

The pooled analysis demonstrated that the incidence of temporary facial palsy was comparable between the overall LS and CT groups (RD, 0.00; 95% CI, −0.03 to 0.04, *I*^2^ = 0.00%) (Figure 4A). Subgroup analysis including the three studies using LSP [12,14,15] showed that the incidence of temporary facial palsy was comparable between the LSP group and the CT group (RD: 0.00; 95% CI, −0.10 to 0.10, *I*^2^ = 0.00%) (Figure 4B). Subgroup analysis including the two studies using LSJ [11,13] also revealed that the incidence of temporary facial palsy was comparable between the LSJ group and the CT group (RD: 0.06; 95% CI, −0.12 to 0.24, *I*^2^ = 62.24%) (Figure 4C). One of the five studies included deep lobe parotid tumors in their report, and there was no significant intergroup difference regarding the incidence of permanent and temporary facial palsy after removing this study from the analysis (RD, −0.01; 95% CI, −0.07 to 0.05, *I*^2^ = 0.00%, and RD, 0.03; 95% CI, −0.07 to 0.13, *I*^2^ = 15.08%, respectively) (Figure S1B,C of the Supplementary Material). Random effects meta-analysis was also calculated with the only one randomized controlled trial excluded [14]. There were no marked differences in the direction or significance of our findings when this study was excluded (Figure S2B,C of the Supplementary Material).

#### 3.3.3. Postoperative Salivary Complications (Salivary Fistula/Seroma)

**Figure 5:**
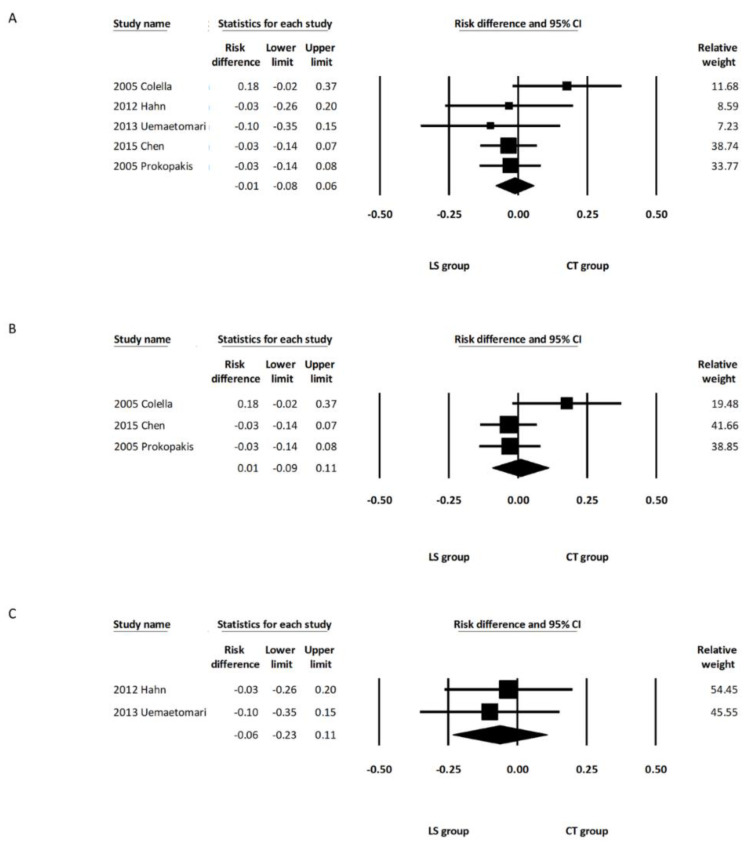
Forest plot of the incidence of salivary complications after parotidectomy. (**A**) Overall study group. (**B**) Studies including only the LSP device. (**C**) Studies including only the LSJ device. LS, LigaSure; LSP, LigaSure Precise; LSJ, LigaSure Small Jaw; CT, conventional technique; CI confidence interval.

All five studies [11,12,13,14,15] recorded the incidences of salivary complications in both groups. The pooled analysis demonstrated that the incidence of salivary complications was comparable between the overall LS and CT groups (RD, −0.01; 95% CI, −0.08 to 0.06, *I*^2^ = 7.22%) (Figure 5A). Subgroup analysis including the three studies using LSP [12,14,15] revealed a similar rate of salivary complications between the LSP group and the CT group (RD, 0.01; 95% CI, −0.09 to 0.11, *I*^2^ = 47.14%) (Figure 5B). Subgroup analysis including the other two studies using LSJ [11,13] also showed a similar rate of salivary complications between the LSJ group and the CT group (RD, −0.06; 95% CI, −0.23 to 0.11, *I*^2^ = 0.00%) (Figure 5C). One of the five studies included deep lobe parotid tumors in their report, and there was no significant intergroup difference regarding the incidence of salivary complications after removing this study from the analysis (RD, −0.00; 95% CI, −0.08 to 0.08, *I*^2^ = 21.73%) (Figure S1D of the Supplementary Material). A random effects meta-analysis was also calculated with the only one randomized controlled trial excluded [14]. There were no marked differences in the direction or significance of our findings when this study was excluded (Figure S2D of the Supplementary Material).

#### 3.3.4. Postoperative Bleeding Complications

**Figure 6:**
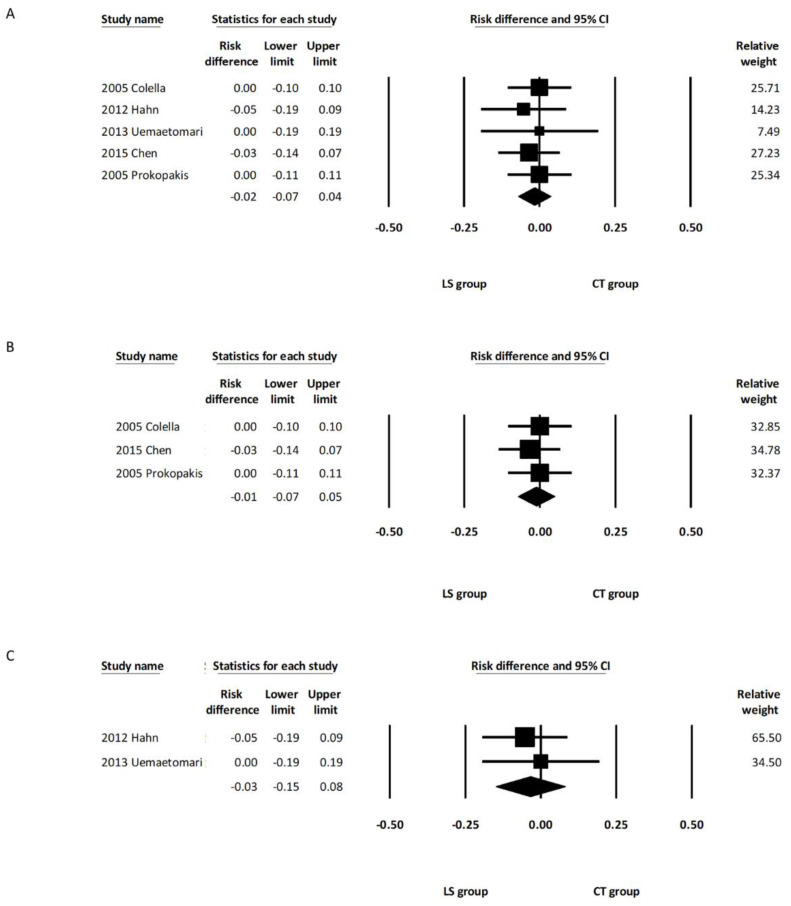
Forest plot of the incidence of bleeding complications after parotidectomy. (**A**) Overall study group. (**B**) Studies including only the LSP device. (**C**) Studies including only the LSJ device. LS, LigaSure; LSP, LigaSure Precise; LSJ, LigaSure Small Jaw; CT, conventional technique; CI confidence interval.

All five studies [11,12,13,14,15] reported incidences of postoperative bleeding. The pooled analysis demonstrated that the incidence of postoperative bleeding was comparable between the overall LS and CT groups (RD, −0.02; 95% CI, −0.07 to 0.04, *I*^2^ = 0.00%) (Figure 6A). Subgroup analysis including the three studies using LSP [12,14,15] revealed a similar rate of postoperative bleeding between the LSP group and the CT group (RD, −0.01; 95% CI, −0.07 to 0.05, *I*^2^ = 0.00%) (Figure 6B). Subgroup analysis including the other two studies using LSJ (11, 13) also showed a similar rate of postoperative bleeding between the LSJ group and the CT group (RD, −0.03; 95% CI, −0.15 to 0.08, *I*^2^ = 0.00%) (Figure 6C). One of the five studies included deep lobe parotid tumors in their report, and there was no significant intergroup difference regarding the incidence of bleeding complications after removing this study from the analysis (RD, −0.02; 95% CI, −0.07 to 0.04, *I*^2^ = 0.00%) (Figure S1E of the Supplementary Material). Random effects meta-analysis was also calculated with the only one randomized controlled trial excluded [14]. There were no marked differences in the direction or significance of our findings when this study was excluded (Figure S2E of the Supplementary Material).

### 3.4. Publication Bias

Heterogeneity tests and funnel plots are displayed in Table S5 of the Supplementary Material. Egger’s test of the operative duration was positive in the overall study group (*p* = 0.01), suggesting that publication bias may have an impact on the results. According to Egger’s test, no apparent publication bias was noted among other parameters.

## 4. Discussion

According to the present meta-analysis, the LS group had a shorter operative time in parotidectomy, which was significantly different from the CT group. In addition, the incidences of postoperative facial palsy, salivary complications, and bleeding complications did not show significant differences between the two groups. To our knowledge, this is the first study that systemically reviewed and compared these two techniques in parotidectomy.

Bipolar vessel-sealing devices developed to facilitate surgery were first introduced in 1998 [17]. The devices grasp tissue bundles or vessels, compress them, and apply voltage between bipolar forceps. The flow of the current creates a thermal effect that causes the sealing of vessel walls [18]. Most tissue proteins denature at the desired temperature of 60 to 70 °C. Because the thermal energy is mostly confined within the forceps, there is minimal collateral thermal injury [18]. LS is a bipolar vessel-sealing instrument that has been used in laparoscopic and open procedures in various surgical specialties [9,10,19]. It has also been utilized in head and neck surgery in the last two decades [8,10,20]. In the reported literature, LSP and LSJ are the two types of LS most commonly used in parotidectomy. The LSP is a single-patient instrument with a 16.5 cm long, 15-degree jaw angle handpiece, and a foot-switching pedal. The LSP was able to seal vessels and provide reliable hemostasis. However, surgical scissors were required to transect the sealed tissue after LSP application [21]. On the other hand, the LSJ is an 18.8 cm long single-use instrument with a 28-degree jaw angle and a tactile feedback activation button. It can be triggered by either a handpiece or foot pedal [22]. Unlike LSP, LSJ is a multifunctional design that incorporates a cutting device in addition to a vessel sealing system. Surgeons were able to cut the sealed tissue immediately after the sealing process.

The LS device has been applied in various otolaryngology head and neck surgeries for years. A search in the previous literature revealed that the LS group had a significantly shorter operative time than the conventional group in surgeries including oral cancer excision, neck dissection, thyroidectomy, laryngectomy, tonsillectomy, and submandibular and parapharyngeal tumor excision [8,10,20,23]. Our meta-analysis showed that the operative time in parotidectomy was significantly decreased in the overall LS group. Subgroup analysis also revealed that the operative time was shorter in either the LSP or LSJ group than in the CT group. The shorter operative time may be attributed to faster hemostasis without the conventional time-consuming clamp-and-tie procedure. This result is compatible with the previous literature, showing that the LS group had a significant reduction in operative time compared to the conventional group in parotid surgery.

Facial palsy is one of the most common complications of parotidectomy. The documented incidences of temporary facial nerve palsy and permanent facial palsy following parotidectomy were 15% to 66% and 2.5% to 5.0%, respectively [24,25]. Facial palsy is not only a physical movement disorder but also has a much more profound impact on patients because it may lead to psychosocial difficulties, including anxiety, depression, poor social functioning, and low quality of life [26]. As a result, preservation of the facial nerve and avoidance of facial palsy have been critical and prioritized in surgical procedures for parotidectomy. Our study compares the pooled incidence of both temporary and permanent facial nerve palsy between parotidectomies in the LS and CT groups. The results demonstrated that the risk of temporary or permanent facial nerve palsy was comparable between the two groups, indicating that the use of LS in parotid surgery does not increase the risk of facial nerve injury.

Salivary fistula or seroma is reported to occur in 4% to 39% of postparotidectomy patients [27]. Previous studies have suggested that salivary fistula or seroma formation may be related to the use of Surgicel within the wound bed [28]. However, the use of Surgicel was not reported in the studies included in our study, which makes further analysis impossible. Our study suggests that the use of LS does not increase the risk of postoperative salivary fistula or sialocele formation. Postoperative bleeding is a relatively rare complication after parotidectomy. Its incidence was reported to be approximately 1% to 5% in previous studies [25,29]. Postoperative bleeding is usually related to inadequate hemostasis during the surgical procedure. Our study showed that there was no significant difference in the postoperative bleeding rate between the LS group and the CT group, meaning that LS did not seem to increase or decrease the bleeding rate after parotidectomy.

There are potential limitations in this study. First, our meta-analysis only included five related studies. In addition, only one of the included studies was a randomized control trial. Third, there was potential publication bias in the operative time parameter in our meta-analysis, which needs to be interpreted with caution. Despite all the limitations, the present study provides evidence of the differences between the use of LS or CT in parotidectomy according to the literature.

## 5. Conclusions

Compared with conventional techniques, LS significantly reduces the operative time needed for parotidectomies. The incidences of postoperative complications, including facial nerve palsy, postoperative salivary complications, and bleeding complications, were comparable between the two groups. Surgeons may consider using LS according to cost-effectiveness and personal preferences. The reduction in operative time may be more beneficial, especially for those with a higher risk from general anesthesia.

## Figures and Tables

**Table 1:** Characteristics of the included studies.

Authors	Year	Country	Study Design	Number of Patients		Mean Age (Years)		Gender (M/F)		Surgery Type	LigaSure Type	Final Diagnosis
				LS	CT	LS	CT	LS	CT			
Colella et al.	2005	Italy	Prospective	17	18	45	49	8/9	7/11	SP	LSP	PA, WT, LP, MY
Prokopakis et al.	2005	Greece	Prospective	12	103	51	54	3/9	24/79	SP	LSP	N/A
Hahn et al.	2013	Denmark	Prospective	16	19	59	53	6/10	12/7	SP	LSJ	PA, WT, ON, SA, LC
Uemaetomari et al.	2013	Japan	Retrospective	8	10	54.5	50.4	N/A	N/A	SP, TP	LSJ	PA, WT
Chen et al.	2015	Taiwan	Prospective	20	29	51.6	51.4	13/7	21/8	SP	LSP	PA, WT, OTH

PA, Pleomorphic adenoma; WT, Warthin’s tumor; LP, Lipoma; MY, Myoepithelioma; ON, Oncocytoma; SA, Sarcoidosis; LC, Lymphoepithelial cyst; OTH, Others; N/A, Not available; SP, Superficial parotidectomy; TP, Total parotidectomy; LS, LigaSure; CT, Conventional technique; LSP, LigaSure Precise; LSJ, LigaSure Small Jaw.

## Data Availability

The data presented in this study are available in the included studies or Supplementary Material.

## Supplementary Materials

The following supporting information can be downloaded at: https://www.mdpi.com/article/10.3390/healthcare10040706/s1, Table S1: Literature Searches and Keywords; Table S2: Newcastle–Ottawa Scale Quality Assessment of Included Nonrandomized Studies; Table S3: Risk of Bias Assessment of Included Randomized Studies; Table S4: PRISMA checklist; Table S5: Funnel plots; Figure S1: Forest Plots of Studies of Superficial Parotidectomy; Figure S2: Forest Plots of Studies of Observational Studies.
